# Apigenin induces oxidative stress in mouse Sertoli TM4 cells

**DOI:** 10.14202/vetworld.2021.3132-3137

**Published:** 2021-12-20

**Authors:** Sathaporn Jittapalapong, Thapanee Poompoung, Samak Sutjarit

**Affiliations:** Department of Veterinary Technology, Faculty of Veterinary Technology, Kasetsart University, Bangkok 10900, Thailand

**Keywords:** apigenin, malondialdehyde, oxidative stress, reactive oxygen species, TM4 cells

## Abstract

**Background and Aim:**

Apigenin (API) is an estrogenic compound found in many plants. Sertoli cells reside in the testis and are a key target of environmental toxicants. This study aimed to examine the cytotoxicity, especially oxidative stress of API in mouse Sertoli TM4 cells.

**Materials and Methods:**

Mouse Sertoli TM4 cells were treated with 50 and 100 μM API for 48 h. Cell viability, lactate dehydrogenase (LDH) activities, glutathione reductase (GR) activities, production of reactive oxygen species (ROS), and malondialdehyde (MDA) levels were evaluated using various assays.

**Results:**

Treatment with API at both 50 and 100 μM decreased viability and GR activity but increased LDH activity, ROS production, and MDA levels in mouse Sertoli TM4 cells.

**Conclusion:**

Exposure to API induced oxidative stress in mouse Sertoli TM4 cells.

## Introduction

Over the past few decades, bioactive compounds in plants have attracted increasing interest from the public and scientific community due to their vast therapeutic benefits [1-3]. Notably, pharmacological studies have discovered that 4’,5,7,-trihydroxyflavone or apigenin (API), an estrogenic flavonoid found abundantly in oranges, grapefruit, onions, celery, parsley, wheat sprouts, green tea, chamomile, spearmint, and thyme [4-13], possesses anti-inflammatory [9,14-21], antioxidant [8,9,17,19,21], antimicrobial [14-16,18,20], anticancer [9,14-18,20,22], hepatoprotective [23], renoprotective [8], and neuroprotective [24] properties.

In contrast, emerging evidence has shown that Sertoli cells are particularly sensitive to harmful substances in food [25], hormonal treatments [25-27], and environmental toxicants. These cells are located in the seminiferous tubules of the testis and provide nutrients and structural support for developing spermatids [28,29]; therefore, disrupting them through xenobiotic-induced toxicity might compromise male reproductive health and function [30].

To the best of our knowledge, some studies have reported the effects of phytoestrogen on male reproduction. However, information about cytotoxic effects, especially oxidative stress of API is scarce. This study aimed to examine the cytotoxicity, especially oxidative stress of API in mouse Sertoli TM4 cells.

## Materials and Methods

### Ethical approval

No experiments in this study were conducted on live animals, so ethical approval was unnecessary.

### Study period and location

The study was conducted from March to December 2020 at the Department of Veterinary Technology, Kasetsart University, Thailand.

### Cells and chemicals

Mouse Sertoli TM4 cells were purchased from the American Type Culture Collection (Manassas, VA, USA). MTS cell proliferation assay kit, lactate dehydrogenase (LDH)-cytotoxicity assay kit II, 2’7’-dichlorofluorescin diacetate (DCFDA) cellular reactive oxygen species (ROS) detection assay kit, lipid peroxidation assay kit (malondialdehyde [MDA]), and GR assay kit were purchased from Abcam (Cambridge, MA, USA). API was purchased from Sigma-Aldrich (St. Louis, MO, USA) and mixed with dimethyl sulfoxide solution (DMSO) before being filtered.

### Cell culture

Mouse Sertoli TM4 cells were cultured in complete Dulbecco’s Modified Eagle Medium/F-12 supplemented with 5% horse serum, 2.5% fetal bovine serum, 100 U/μL penicillin, and 100 U/μL streptomycin in a humidified atmosphere with 5% CO_2_ at 37°C.

### MTS assay

Cell viability was determined using the MTS cell proliferation assay kit. Briefly, mouse Sertoli TM4 cells were seeded into 96-well plates at a density of 5-10×10^3^ cells/well at a final volume of 200 μL/well and incubated for 24 h. Next, the cells were treated with API at concentrations of 0, 20, 40, 60, 80, 100, 120, 140, and 160 μM or DMSO. In this test, we used two controls: Zero concentration of API and DMSO. The result showed DMSO has no influence on cells. However, we calculated the results between the zero concentration and API treatment groups. Then, the cells were further incubated for 24, 48, and 72 h in a humidified atmosphere with 5% CO_2_ at 37°C. After this incubation, 20 μL MTS reagent was added to each well, and the cells were further incubated for 1-2 h at 37°C in the dark. Finally, absorbance was measured using an automated microplate reader (Bio-Rad, USA) at 490 nm. The results were expressed as a percentage of viable cells compared with the control.

### LDH leakage assay

LDH leakage was measured using an LDH cytotoxicity assay kit. Briefly, mouse Sertoli TM4 cells were seeded into 96-well plates at a density of 5-10×10^3^ cells/well at a final volume of 200 μL/well. After cells became attached to the well, the medium was replaced with 100 μL medium containing 50 and 100 μM API, and then, the cells were incubated for 48 h. At the end of incubation, the plate was gently shaken to ensure that LDH was evenly distributed in the culture medium. Next, the cells were centrifuged at 600×*g* for 10 min, and the precipitate was transferred into new 96-well plates (10 μL/well). Then, 100 μL LDH reaction mix was added to each well, and the plate was incubated for 60 min at 25°C. After incubation, the absorbance was measured using an automated microplate reader (Bio-Rad) at 450 nm.

### Determination of intracellular ROS production

ROS was measured using a DCFDA cellular ROS detection kit. Briefly, mouse Sertoli TM4 cells were seeded into 96-well plates at a density of 5-10×10^3^ cells/well at a final volume of 200 μL/well. After the cells became attached to the well, the medium was replaced with 100 μL medium containing 50 and 100 μM API, and then, the cells were incubated for 48 h. Next, each well was washed with 100 μL 1×buffer and stained with 100 μL 25 μM DCFDA in 1×buffer for 45 min at 37°C in the dark. Finally, this solution was removed from the cells, and 100 μL 1×buffer was added to each well for measuring fluorescence at Ex/Em=485/535 nm in endpoint mode.

### Determination of lipid peroxidation

Lipid peroxidation was measured using an MDA assay kit. Briefly, mouse Sertoli TM4 cells were seeded into 96-well plates at a density of 5-10×10^3^ cells/well at a final volume of 200 μL/well. After the cells became attached to the well, the medium was replaced with 100 μL medium containing 50 and 100 μM API, and then, the cells were incubated for 48 h. Next, thiobarbituric acid was added to the wells, and the cells were incubated for 60 min at 95°C. Finally, the cells were cooled in an ice bath for 10 min, and the absorbance was measured using an automated microplate reader (Bio-Rad) at 532 nm.

### Determination of GR activity

GR activity was measured using a GR assay kit. Briefly, mouse Sertoli TM4 cells were seeded at 1-5×10^5^ cells and treated with 50 and 100 μM API or nothing for 48 h before being homogenized with 0.2 μL cold assay buffer. The cells were centrifuged at 10,000×*g* for 15 min at 4°C, and then, the supernatant was collected and stored at −80°C until further testing. Before performing the GR assay, the samples were treated with 3% H_2_O_2_ and catalase to destroy glutathione (GSH). Then, the samples were seeded into 96-well plates and mixed with 50 μL reaction mixture. The absorbance was measured using an automated microplate reader (Bio-Rad) at 405 nm.

### Statistical analysis

All experiments were repeated at least thrice, and values are expressed as mean±standard deviation. Paired t-tests were employed to determine when differences between values were statistically significant, with p<0.05 regarded as statistically significant.

## Results

### Effect of API on the viability of TM4 cells

**Figure-1:**
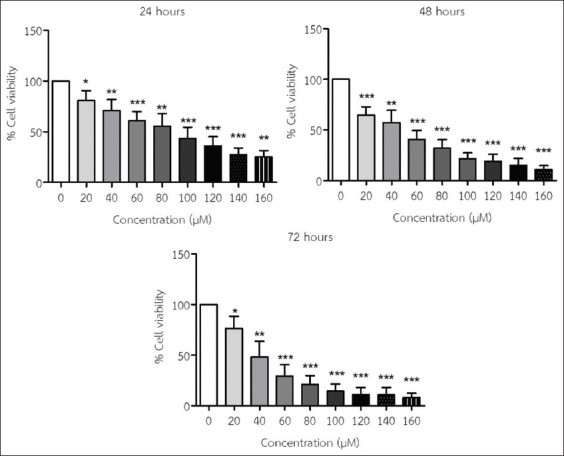
TM4 cell viability was determined using an MTS assay after incubation with varying concentrations of apigenin for 24, 48, and 72 h (mean±SD). *p<0.05; **p<0.005; and ***p<0.001 compared with no treatment. All results are the mean values obtained from at least three independent experiments.

The results indicated that API was cytotoxic to TM4 cells in a time- and concentration-dependent manner, as the half-maximal inhibitory concentration (IC_50_) values were 95.5, 47.7, and 40.9 μM at 24, 48, and 72 h of incubation, respectively (Figure-1). According to our review of literature, we used the API concentrations of 0-100 μM for 24-114 h to test human hepatoma cells and human leukemia cell lines [31,32]. Moreover, note that 48 h was selected as the optimal time because the IC_50_ value was not high, and the concentrations of API at 50 and 100 μM were used in later experiments.

### Effect of API on LDH activity in TM4 cells

**Figure-2:**
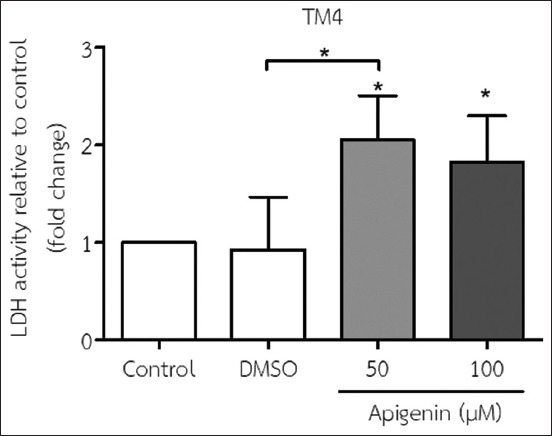
Lactate dehydrogenase (LDH) activity in TM4 cells was determined using an LDH cytotoxicity assay after incubation with 50 and 100 μM apigenin for 48 h (mean±SD). *p<0.05 compared with no treatment (the control) and dimethyl sulfoxide treatment. All results are the mean values obtained from at least three independent experiments.

The findings showed that the addition of 50 and 100 μM API to the medium significantly increased LDH activity in TM4 cells compared with no treatment (control). In addition, a striking increase in LDH activity was observed in cells treated with 50 μM API relative to DMSO (Figure-2).

### Effect of API on ROS production in TM4 cells

**Figure-3:**
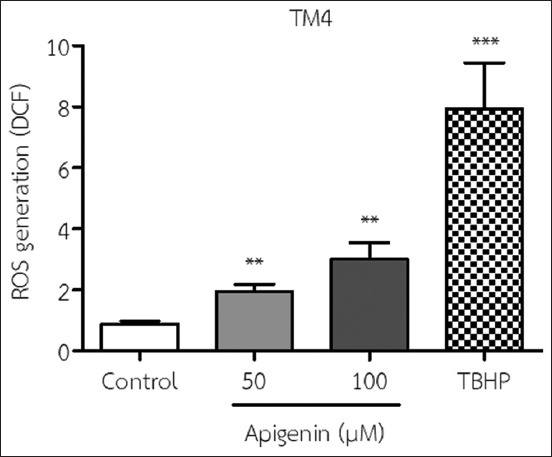
Reactive oxygen species (ROS) production in TM4 cells was determined using an ROS detection assay after incubation with 50 and 100 μM apigenin for 48 h (mean±SD). Tert-butyl hydroperoxide is positive control. **p<0.005 and ***p<0.001 compared with no treatment (the control). All results are the mean values obtained from at least three independent experiments.

The results showed that the addition of 50 and 100 μM API to the medium significantly increased ROS levels in TM4 cells compared with no treatment (control) (Figure-3).

### Effect of API on MDA levels in TM4 cells

**Figure-4:**
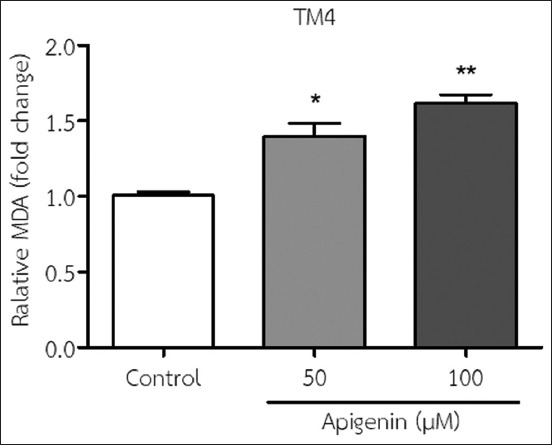
Malondialdehyde (MDA) levels in TM4 cells were determined using an MDA assay after incubation with 50 and 100 μM apigenin for 48 h (mean±SD). *p<0.05 and **p<0.001 compared with no treatment (the control). All results are the mean values obtained from at least three independent experiments.

The findings indicated that the addition of 50 and 100 μM API to the medium significantly increased MDA levels in TM4 cells compared with no treatment (control) (Figure-4).

### Effect of API on GR activity in TM4 cells

**Figure-5:**
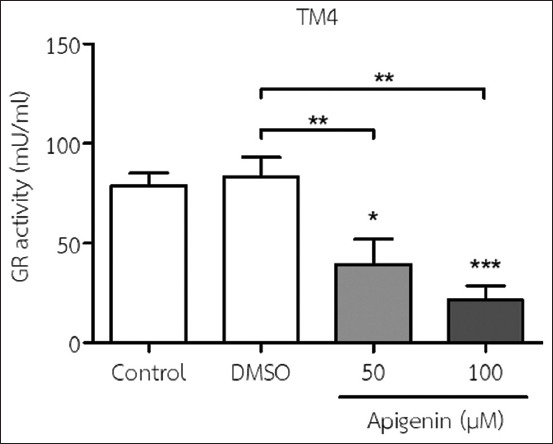
Glutathione reductase (GR) activity in TM4 cells was determined using a GR assay after incubation with 50 and 100 μM apigenin for 48 h (mean±SD). *p<0.05; **p<0.01; and ***p<0.001 compared with no treatment (the control) and dimethyl sulfoxide treatment. All results are the mean values obtained from at least three independent experiments.

The results showed that the addition of 50 and 100 μM API to the medium significantly decreased GR activity in TM4 cells compared with no treatment (control) and DMSO treatment (Figure-5).

## Discussion

In this investigation, mouse Sertoli TM4 cells exposed to various concentrations of API experienced significant increases in oxidative stress. Cytotoxicity assays are advantageous for determining a compound’s potential to invoke apoptosis (i.e., cell death) [33]. Here, LDH leakage and MTS assays were used, which are superior to MTT assays because their reagents are added directly to the culturing medium without multiple steps. From these kits, analyses demonstrated that API was cytotoxic to TM4 cells in a time-dependent manner. Copious research has established that API increases LDH activity levels in TM4 cells while decreasing the proliferation of numerous cell lines, such as human hepatocarcinoma HepG2 cells [31], human leukemia [32], human hepatocarcinoma Hep3B and HepG2 [34], human cholangiocarcinoma [35], and human breast carcinoma [36].

Oxidative stress result from an imbalance between the production and accumulation of ROS inside cells and an individual’s inability to detoxify ROS. Hence, ROS generation and increased lipid peroxidation are signs of oxidative stress [37]. In this study, we found that the addition of API to the culturing medium significantly increased ROS levels in TM4 cells, which agrees with the findings from comparable investigations that exposure to API elevates ROS levels in human hepatocarcinoma HepG2 cells [31], human leukemia [32], human hepatocarcinoma Hep3B and HepG2 [34], and human breast carcinoma [36]. Furthermore, our data revealed that API increased MDA levels (the end product of oxidative injury and an indicator of lipid peroxidation) and decreased GR activity (an enzyme involved in the GSH redox cycle) in TM4 cells. According to our review of literature, we reported that API-induced GR activity inhibits ethanol-induced oxidative stress and lipopolysaccharide (LPS)-induced inflaμMatory cytokine production in cultured rat hepatocytes [38]; API-induced GR activity inhibits d-galactosamine/LPS-induced liver injury by upregulating hepatic Nrf-2 and peroxisome proliferator-activated receptor g expressions in mice [39]; GR activity has antioxidant properties and superoxide dismutase and GR activities in HepG2 cells after the addition of a fungal endophyte producing API from pigeon pea (*Cajanus cajan* (L.) Millsp.) [40]. In this study, we used a GR assay kit because GR catalyzes the nicotinamide adenine dinucleotide phosphate-dependent reduction of the oxidized GSH (a non-enzymatic antioxidant) to reduce GSH ratio, which plays an important role in the GSH redox cycle. A high oxidized GSH/reduced GSH ratio is essential for protection against oxidative stress.

This study has strengths and limitations that should be mentioned. Regarding study strengths, mouse Sertoli TM4 cells were selected because they share features with human Sertoli cells [41] and are useful in evaluating the impact of API exposure *in* *vitro*. The main disadvantage of an *in vitro* study is the lack of systemic effects, while that of an *in vivo* study is the animal welfare legislation, which is currently applicable in most countries to prevent misuse of animals and decrease the number of animals required in toxicological testing. Hence, Sertoli cells are essential for spermatogenesis and are a major target for various toxicants; thus, they serve as an ideal model for toxicity studies on the male reproductive system.

## Conclusion

This study demonstrated that exposure to API induces oxidative stress in mouse Sertoli TM4 cells, as evidenced by reductions in cell viability and GR activity and increases in LDH activity, ROS production, and MDA levels.

## Authors’ Contributions

SS and SJ: Conceptualization. SS and TP: Methodology and laboratory experiments. SS, TP, and SJ: Interpretation of the data. SS: Writing of the manuscript. All authors have read and approved the final manuscript.
